# Quality of Life and Related Factors in Children With Dystonia

**DOI:** 10.1002/brb3.71476

**Published:** 2026-05-14

**Authors:** Kamile Uzun Akkaya, Esra Serdaroglu, Pelin Atalan Efkere, Zekiye Basaran, Bulent Elbasan

**Affiliations:** ^1^ Department of Physiotherapy and Rehabilitation, Faculty of Health Sciences Gazi University Ankara Turkey; ^2^ Department of Pediatric Neurology, Faculty of Medicine Gazi University Ankara Turkey

**Keywords:** child, constipation, dystonia, functional independence, quality of life

## Abstract

**Background:**

Dystonia is characterized by continuous involuntary contractions of agonist and antagonist muscles, adversely affecting functionality. It is among the most prevalent forms of movement disorders observed during childhood. We aimed to measure dystonia severity, motor functions, sleep quality, constipation, and functional independence in children with dystonia, and to examine the relationships between these parameters and quality of life in this exploratory study.

**Methods:**

Children's dystonia severity, motor function, sleep disorders, constipation, independence in daily activities, and quality of life were assessed using the Burke–Fahn–Marsden Dystonia Rating Scale, Gross Motor Function Measure‐88, Sleep Disturbance Scale for Children, Constipation Assessment Scale, WeeFIM, and Caregiver Priorities and Child Health Index of Life with Disabilities (CPCHILD) parent form, respectively. The relationship between dystonia severity, motor functions, sleep disorders, constipation problems, independence in daily living activities and quality of life was examined.

**Results:**

A total of 21 children aged 4–18 years with dystonia were included in the study. The results indicated that dystonia severity, motor functions, constipation status, and levels of functional independence were associated with quality of life (*p* < 0.05), whereas sleep status was not associated (*p* > 0.05).

**Conclusions:**

In children with dystonia, several parameters, such as dystonia severity, motor functions, constipation, and the level of independence in daily living activities, may be associated with quality of life. These findings may help inform future research and may be relevant when considering intervention priorities.

## Graphical abstract

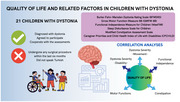

## 1 Introduction

Movement disorders in childhood are conditions in which the child is unable to perform the desired movement, unable to suppress involuntary movements, or experiences both simultaneously. Movement disorders in children are classified into two groups: hyperkinetic and hypokinetic movement disorders. Dystonia is a hyperkinetic movement disorder and one of the most common movement disorders during childhood, with a prevalence of approximately 2–50 per million for early‐onset cases (< 26 years) and 30–7320 per million for late‐onset (> 26 years) dystonia (Blackburn and Parnes 2021; Fernández‐Alvarez and Nardocci 2012; Singer et al. 2015). According to the latest consensus, dystonia is a hyperkinetic movement disorder characterized by sustained or intermittent involuntary muscle contractions that result in abnormal, often repetitive movements and postures. These motor manifestations are typically patterned and stereotyped, and may present with tremulous or jerky features. Symptoms are frequently precipitated or exacerbated by voluntary movement and are commonly associated with overflow muscle activation beyond the intended motor task (Albanese et al. 2025). Dystonia may arise from a genetic disorder or present as a symptom of an underlying condition such as brain injury, encephalitis, vascular diseases, autoimmune diseases, cerebral malformations, metabolic disorders, or neurodegenerative diseases (Meijer and Pearson 2018). Dystonia classifications are being made according to age (early‐onset and late‐onset), distribution (focal, segmental, multifocal, generalized, and hemidystonia) or etiology (primary and secondary) (Defazio 2006).

Disability‐driven and dystonia‐specific quality of life are the two main perspectives of quality of life in patients with dystonia. The overall impact of functional limitations arising from disability reflects the disability‐driven quality of life. The effects of involuntary movements, abnormal postures, pain and social‐emotional restrictions, which are motor and non‐motor symptoms of dystonia, form the perspective captured by dystonia‐specific quality of life. These manifestations affect independence in activities of daily living, hence impair both the child's and the family's quality of life. Even though these perspectives on quality of life seem quite similar, they represent the different aspects of disease burden. That's why they should be considered separately. In this study, quality of life was assessed by Caregiver Priorities and Child Health Index of Life with Disabilities‐Caregiver Form (CPCHILD). It reflects disability‐driven quality of life and is validated for children with disabilities. There is a lack of validated measurement tools assessing the dystonia‐specific quality of life and CPCHILD provides an assessment of overall functional impact, although it may not fully capture the disease‐specific burden of dystonia (Eggink et al. 2019; Jain et al. 2021; Narayanan et al. 2006; Roubertie et al. 2012).

While numerous studies in the literature have examined quality of life in adults with dystonia, the number of studies conducted in children remains limited (Girach et al. 2019; Tomic et al. 2016; Zetterberg et al. 2009). Although most studies have reported that quality of life is generally negatively affected in children with dystonia (Eggink et al. 2019; Monbaliu et al. 2017), one study concluded that their quality of life was comparable to that of their healthy peers (Timmers et al. 2017). In current studies, motor and non‐motor aspects such as motor skills, quality of life, depression, anxiety, and sleep have been evaluated separately in children with dystonia (Peall et al. 2023; Zhou et al. 2024). Gastrointestinal problems, especially constipation—one of the non‐motor problems, are commonly seen both in children with neurodevelopmental disorders and in individuals with various movement disorders (Sakakibara 2021; Sullivan 2008). However, it is noteworthy that constipation problems were not investigated in children with dystonia in the studies conducted. Determining motor and non‐motor factors that influence quality of life in children with dystonia will be important for identifying intervention strategies aimed at improving their quality of life. This exploratory study aims to examine the relationships between quality of life and dystonia severity, motor functions, sleep, constipation, and functional independence levels in children diagnosed with dystonia.

## 2 Methods

### 2.1 Participants

This exploratory study was performed at the Pediatric Rehabilitation Unit of the Department of Physiotherapy and Rehabilitation, Gazi University, between April 2022 and March 2024. Children diagnosed with dystonia by a pediatric neurologist at the Department of Pediatric Neurology, Faculty of Medicine, Gazi University, who met the inclusion criteria, were enrolled in the study. Signed informed consent was obtained from the parents of all participating children. Ethical approval for the study was granted by the Ethics Committee of Gazi University (approval no: E‐77082166‐604.01.02‐300406) on February 22, 2022.

The study included children aged 4–18 years with a confirmed diagnosis of dystonic movement disorder who agreed to participate in the evaluations and were able to cooperate sufficiently with the clinical assessments. Children who had undergone Botulinum toxin injections or any surgical procedure within the last 6 months, as well as those with parents who did not speak Turkish, were excluded.

### 2.2 Assessments

First, demographic information such as age, height, and body weight was recorded. The etiology (inherited, acquired), distribution (generalized, focal), and type (primary, secondary) of dystonia were investigated. Subsequently, dystonia severity, motor functions, sleep disorders, constipation problems, independence in daily living activities, and quality of life were assessed. Assessments were scheduled according to ongoing treatments (e.g., before daily medications, at least 24 h after the last physiotherapy session) to minimize the acute treatment effects on outcome measures. They were conducted in a quiet room, with children accompanied by their parents, to ensure a comfortable and standardized environment.

#### 2.2.1 Assessment of Dystonia Severity

Dystonia severity in children was assessed using the Burke–Fahn–Marsden Dystonia Rating Scale (BFMDRS). The BFMDRS, which is used to evaluate dystonia severity, consists of two subscales: the Movement Scale (BFMDRS‐MS) and the Disability Scale (BFMDRS‐DS). In the BFMDRS‐MS section, dystonia severity and provoking factors are evaluated across nine different body regions (eyes, mouth, speech or swallowing, neck, arms [right and left], trunk, and legs [right and left]). The BFMDRS‐DS section assesses the individual's level of dependence during daily activities such as speaking, writing, feeding, eating, swallowing, hygiene, dressing, and walking. A video recording protocol lasting less than 5 min was applied to all participants. The BFMDRS has an acceptable validity and inter‐ and intra‐rater reliability in dystonia (Burke et al. 1985; Krystkowiak et al. 2007).

#### 2.2.2 Assessment of Motor Functions

The Gross Motor Function Measure‐88 (GMFM‐88) was used to evaluate children's motor function. The GMFM‐88 consists of five subdimensions: lying and rolling, sitting, crawling and kneeling, standing, walking, running, and jumping. It includes 88 items related to motor performance (Russell et al. 1989).

#### 2.2.3 Assessment of Sleep

Sleep disturbances in children were assessed using the Sleep Disturbance Scale for Children, which was completed by the parents. The scale consists of 26 items, is based on a Likert‐type format, and includes six subscales (Bruni et al. 1996). In this study, the Turkish version validated and tested for reliability by Ağadayı et al. (2020) was used. As this assessment relies on parent‐reported data, it may be subject to reporting bias.

#### 2.2.4 Assessment of Constipation

Constipation in children was assessed using the Modified Constipation Assessment Scale, a valid and reliable tool consisting of nine items. The total score ranges from 0 (no constipation) to 18 (severe constipation) (McMillan and Williams 1989; Tarsuslu et al. 2009).

#### 2.2.5 Assessment of Activities of Daily Living

The Functional Independence Measure for Children (WeeFIM) was employed to determine independence in daily life activities among children and adolescents. The WeeFIM comprises six domains—self‐care, sphincter control, mobility, locomotion, communication, and social interaction—and includes a total of 18 items. Each item is scored on a scale from 1 to 7, based on whether the individual requires assistance, completes the function in a timely manner, or needs assistive devices (Msall et al. 1996; Tur et al. 2009).

#### 2.2.6 Assessment of Quality of Life

Today, no dystonia‐specific quality‐of‐life assessment tool is available for children. Among existing quality‐of‐life measures, the CPCHILD has been widely used to assess quality of life in pediatric disorders. It demonstrates strong psychometric properties that capture areas related to functional limitations and caregiver perspectives (e.g., personal care, mobility, comfort, and emotions) (Carlon et al. 2010). Its use in this study was preferred because of its application in the literature for pediatric movement disorders (Koy et al. 2022; Hagelschuer and Koy 2026). CPCHILD is a reliable and valid measure that reflects parents’ perspectives on their children's health status, functional limitations, and well‐being. It consists of 36 items grouped under the subdomains of personal care/activities of daily living, positioning/transfer/mobility, comfort/emotions, communication/social interaction, health, overall quality of life, and importance of each item to quality of life (Narayanan et al. 2006). The Turkish validity and reliability study was conducted by Şimşek et al. (2019).

### 2.3 Statistical Analysis

The Statistical Package for the Social Sciences (SPSS), version 21.0 (SPSS Inc., Chicago, Illinois, USA) was used for all statistical analyses. The normality of the variables was assessed using visual techniques (histograms and probability plots) as well as statistical tests (Kolmogorov–Smirnov and Shapiro–Wilk). Continuous variables were expressed as mean ± standard deviation, while categorical variables were presented as counts and percentages. Continuous variable associations were evaluated using Pearson or Spearman correlation, depending on the normality of the data. Correlation coefficients were classified as follows: 0–0.24 (weak), 0.25–0.49 (moderate), 0.50–0.74 (strong), and 0.75–1.00 (very strong) (Altman 1990). Given the exploratory nature of the analyses and the number of correlations tested, no formal adjustment for multiple comparisons was applied. Therefore, the reported *p*‐values should be interpreted descriptively, and the findings should not be considered as evidence of independent predictive relationships.

## 3 Results

A total of 21 children diagnosed with dystonic movement disorder were included in the study. Of these, nine (42.9%) were girls, and 12 (57.1%) were boys, with a mean age of 13.33 ± 3.86 years. Demographic data, such as age, height, and weight, are presented in Table 1, while the individual characteristics of each child are provided in Table 2.

**TABLE 1:** Demographic data of the children.

Demographic data	Mean ± SD	Min–Max
**Gestational age (week)**	37.53 ± 4.76	26–41
**Age (year)**	13.33 ± 3.86	5–18
**Height (cm)**	157.68 ± 19.62	88–175
**Weight (kg)**	50.21 ± 15.91	11–70
	**Number (*n*)**	**Percentage (%)**
**Sex** Female Male	9 12	42.9 57.1
**Type of birth** Cesarean section Normal	13 8	61.9 38.1
**Etiology** Inherited Acquired	11 10	52.4 47.6
**Anatomical distribution** Generalized Focal	20 1	95.2 4.8
**Type of dystonia** Primary Secondary	6 15	28.6 71.4

Abbreviations: cm, centimeter; kg, kilogram; Max, maximum; Min, minimum; SD, standard deviation.

**TABLE 2:** Characteristics of study participants.

	Sex	Age (year)	Diagnosis	Etiology	Dystonia distribution	Type of dystonia
Case 1	M	12	KMT2B, genetic	Inherited	Generalized	Primary
Case 2	F	10	Ataxia telangiectasia, ATM gene	Inherited	Generalized	Secondary
Case 3	F	18	CP	Acquired	Generalized	Secondary
Case 4	M	14	CP	Acquired	Generalized	Secondary
Case 5	M	14	KMT2B, genetic	Inherited	Generalized	Primary
Case 6	F	17	PKAN	Inherited	Generalized	Secondary
Case 7	M	14	CP	Acquired	Generalized	Secondary
Case 8	F	8	POLR3A, genetic	Inherited	Generalized	Primary
Case 9	M	14	CP	Acquired	Generalized	Secondary
Case 10	M	5	Hashimoto's encephalopathy	Acquired	Generalized	Secondary
Case 11	F	18	PKAN	Inherited	Generalized	Secondary
Case 12	M	15	CP	Inherited	Generalized	Secondary
Case 13	F	16	PKAN	Inherited	Generalized	Secondary
Case 14	F	12	CP	Acquired	Generalized	Secondary
Case 15	F	16	Genetic	Inherited	Focal	Primary
Case 16	M	13	KMT2B, genetic	Inherited	Generalized	Primary
Case 17	M	15	SLC18A2, genetic	Inherited	Generalized	Secondary
Case 18	M	17	CP	Acquired	Generalized	Secondary
Case 19	M	17	CP	Acquired	Generalized	Secondary
Case 20	M	10	GNAO1, genetic	Inherited	Generalized	Primary
Case 21	F	5	SLC18A2, genetic	Acquired	Generalized	Secondary

Abbreviations: CP, cerebral palsy; F, female; M, male; PKAN, pantothenate kinase‐associated neurodegeneration.

The relationships and correlation values between the BFMDRS movement and disability subscales and the total quality‐of‐life scores are presented in Table 3. A strong negative correlation was observed between BFMDRS‐MS and CPCHILD total scores (*r* = −0.59, *p* < 0.05), and a very strong negative correlation was found between BFMDRS‐DS and CPCHILD total scores (*r* = −0.85, *p* < 0.05).

**TABLE 3:** The relationship between dystonia severity and quality of life.

		CPCHILD personal care/activities of daily living	CPCHILD positioning/transferring/mobility	CPCHILD comfort/emotions	CPCHILD communication/social interaction	CPCHILD health	CPCHILD overall quality of life	CPCHILD total
BFMDRS‐MS	*r*	−0.51^*a^	**−0.56** ^*a^	**−0.58** ^*a^	−0.35^a^	−0.23^a^	**−0.51** ^*a^	**−0.59** ^*a^
	*p*	**< 0.001**	**0.016**	**0.012**	0.15	0.34	**0.03**	**0.01**
	95% CI lower–upper	−0.795 to −0.042	−0.819 to −0.111	−0.828 to −0.139	−0.709 to 0.155	−0.643 to 0.272	−0.795 to −0.044	−0.832 to −0.151
BFMDRS‐DS	*r*	−0.86^*a^	−0.87^*a^	−0.73^*a^	−0.66^*a^	−0.36^a^	−0.53^*a^	−0.85^*a^
	*p*	**< 0.001**	**< 0.001**	**< 0.001**	**0.003**	0.14	**0.02**	**< 0.001**
	95% CI lower–upper	−0.95 to −0.658	−0.953 to −0.675	−0.898 to −0.398	−0.866 to −0.269	−0.716 to 0.142	−0.805 to −0.072	−0.946 to −0.637

Abbreviations: BFMDRS, Burke–Fahn–Marsden Dystonia Rating Scale; BFMDRS‐DS, BFMDRS Disability Scale; BFMDRS‐MS, BFMDRS Movement Scale; CPCHILD, Caregiver Priorities and Child Health Index of Life With Disabilities; *r*, correlation coefficient.

^a^Spearman correlation coefficient.

*p < 0.05 statistically significant correlation.

In this exploratory study, no significant relationship was observed between sleep quality and quality of life (*p* > 0.05). In contrast, constipation was significantly and negatively correlated with the quality‐of‐life total score (*r* = −0.62, *p* < 0.05), while gross motor function showed a strong positive correlation (*r* = 0.81, *p* < 0.05) (Table 4). The results showed significant correlations between the WeeFIM and quality of life total score in children with dystonia (*r* = 0.83, *p* < 0.05) (Table 4). The correlations of the subscales of the CPCHILD are presented in Table 4.

**TABLE 4:** The relationship between sleep, constipation, motor functions, WeeFIM and quality of life.

		CPCHILD personal care/activities of daily living	CPCHILD positioning/transferring/mobility	CPCHILD comfort/emotions	CPCHILD communication/social interaction	CPCHILD health	CPCHILD overall quality of life	CPCHILD total
SDSC	*r*	−0.28^a^	−0.37^a^	0.00^a^	−0.30^b^	−0.001^b^	−0.11^a^	−0.22^a^
	*p*	0.26	0.12	0.98	0.21	0.99	0.65	0.36
	95% CI lower–upper	−0.669 to 0.229	−0.725 to 0.123	−0.474 to 0.483	−0.671 to 0.196	−0.468 to 0.466	−0.561 to 0.386	−0.637 to 0.281
MCAS	*r*	**−0.60** ^*a^	**−0.62** ^*a^	**−0.56** ^*a^	**−0.58** ^*a^	−0.14^b^	−0.26^a^	**−0.62** ^*a^
	*p*	**0.008**	**0.006**	**0.01**	**0.01**	0.57	0.28	**0.005**
	95% CI lower–upper	−0.839 to −0.172	−0.846 to −0.199	−0.822 to −0.12	−0.77 to −0.009	−0.649 to 0.235	−0.66 to 0.244	−0.85 to −0.211
GMFM‐88 total	*r*	0.82^*a^	0.83^*a^	0.67^*a^	0.74^*a^	0.38^a^	0.57^*a^	0.81^*a^
	*p*	**< 0.001**	**< 0.001**	**0.003**	**< 0.001**	0.13	**0.01**	**< 0.001**
	95% CI lower–upper	0.55–0.935	0.582–0.941	0.268–0.875	0.391–0.904	−0.139 to 0.735	0.112–0.831	0.526–0.931
WeeFIM total	*r*	0.84^*a^	0.82^*a^	0.66^*a^	0.70^*a^	0.16^a^	0.39^a^	0.83^*a^
	*p*	**< 0.001**	**< 0.001**	**0.003**	**0.001**	0.51	0.10	**< 0.001**
	95% CI lower–upper	0.61–0.941	0.571–0.934	0.27–0.867	0.339–0.884	−0.342 to 0.595	−0.105 to 0.733	0.592–0.938

Abbreviations: CPCHILD, Caregiver Priorities and Child Health Index of Life With Disabilities; GMFM‐88, Gross Motor Function Measure‐88; MCAS, Modified Constipation Assessment Scale; *r*, correlation coefficient; SDSC, Sleep Disturbance Scale for Children.

^a^Spearman correlation coefficient.

^b^Pearson correlation coefficient.

*p < 0.05 statistically significant correlation.

## 4 Discussion

Quality of life is impaired in children and young adults with dystonia (Eggink et al. 2019). Identifying the factors affecting quality of life in these individuals and planning intervention programs aimed at improving quality of life are of great importance. In this exploratory study, which aimed to examine the relationship between quality of life and parameters such as dystonia severity, motor functions, sleep, constipation, and levels of functional independence in children with dystonia, the findings suggested that dystonia severity, motor functions, constipation status, and levels of functional independence appeared to be associated with quality of life, whereas sleep disturbances were not found to be significantly associated. However, one should consider the potential conceptual overlap between quality of life and functional measures when interpreting these findings. Functional abilities such as motor functions and independence in daily activities are important components of physical well‐being. They are normally reflected in quality‐of‐life assessments (Grilli et al. 2006). Thus, the associations between quality of life, motor function, and functional independence may be partially influenced by shared construct variance. Together with this, quality of life is a multidimensional construct including emotional and social domains. This suggests that these functional measures may not fully capture the broader concept of well‐being. Overall, the findings of this study regarding the quality of life and functional independence measures should be interpreted with caution.

The BFMDRS is one of the scales used to assess dystonia severity in individuals with dystonia. In the literature, it has been frequently employed in patients undergoing deep brain stimulation (Hale et al. 2020); however, there are very few studies investigating the relationship between dystonia severity and quality of life in children with dystonia. In the study by Eggink et al. (2019), the relationships between motor and non‐motor factors and quality of life were examined in young patients with dystonia aged 6–25 years, and it was reported that children and young individuals with dystonia had poorer quality of life. It was concluded that both motor symptoms, including those assessed by the BFMDRS, and non‐motor symptoms affect quality of life in these individuals, with motor symptoms being particularly influential in children with lesional dystonia. These findings may further indicate that non‐motor symptoms play an important role in the phenotypic characterization of dystonia. In our present study, both the movement and disability subscales of the BFMDRS were found to be associated with quality of life. The findings suggest that as dystonia severity and disability levels increased, quality of life may be reduced in children. Although this is an expected result, it provides an important contribution to the literature by highlighting the deterioration in quality of life among children with dystonia.

Sleep is one of the important factors influencing the quality of life. Observational studies demonstrated that sleep disturbances are prevalent among individuals with adult‐onset dystonia, particularly in cases of cervical dystonia with reported rates ranging from 40% to 70%, but evidence from pediatric populations is limited (Bailey et al. 2021; Hertenstein et al. 2016). It was reported that sleep disturbances in individuals with dystonia were potentially associated with quality of life (Hertenstein et al. 2016; Timmers et al. 2017; Wagle Shukla et al. 2016). In a study including both children and adults with dystonia, the lifetime prevalence of daytime sleepiness was significantly higher in adult patients, whereas sleep problems were not observed in the pediatric group. In the study by Zhou et al. (2024), the pediatric‐onset group was reported to exhibit a lower rate of sleep disturbance compared with the adult‐onset group. Furthermore, in the same study, which examined six children with dystonia and four healthy children, quality of life was found to be similar between the two groups (Timmers et al. 2017). In the conducted study, which included a larger sample group of children and adolescents with dystonia, the sleep status was assessed using a questionnaire, and the results suggested that sleep may not be related to quality of life. This result may have occurred because sleep disorders are less common in children. Nevertheless, caution is warranted in interpreting this result. Considering the multidimensional nature of sleep and the wide range of factors influencing it, future research employing more objective methods and involving larger pediatric samples will be valuable for clarifying the findings presented in this study.

Gastrointestinal problems are also frequently observed in children with neurodevelopmental disorders and in individuals with movement disorders, and one such problem is constipation (Sakakibara 2021; Sullivan 2008). In recent years, non‐motor symptoms such as pain, fatigue, sleep disturbances, depression, and anxiety in children and adults with dystonia have become an area of increasing research interest (Peall et al. 2023; Zhou et al. 2024). Based on our review of the literature, the prevalence of constipation in children with dystonia and its relationship with quality of life have not been previously investigated. Constipation complaints in children with dystonia may occur due to factors such as the effects of medications or reduced functionality. In our study, significant relationships were observed between the Modified Constipation Scale scores and both the total and subscale quality‐of‐life scores in children with dystonia. The findings suggest that as constipation worsens, quality of life may be negatively affected. Constipation should be recognized as one of the factors influencing the quality of life in children and adolescents with dystonia, and interventions aimed at improving this condition should be implemented at an early stage. In addition, dietary habits should be carefully reviewed as part of a comprehensive care plan.

Gross motor function skills are observed to be adversely affected in individuals with dystonia. In a study investigating the effects of dystonia and choreoathetosis on activity, participation, and quality of life in children with dyskinetic cerebral palsy, it was concluded that dystonia had a greater impact on gross motor functions and that treatment should primarily focus on reducing dystonia (Monbaliu et al. 2017). Another study found that motor symptoms, including GMFM results, were associated with lower quality of life in children and young adults with dystonia (Eggink et al. 2019). In line with these studies, our findings also may indicate that children with poorer gross motor function skills had lower overall quality of life as well as reduced quality of life across its subdomains. Part of the observed relationship between GMFM and quality of life may be explained by overall functional abilities of children, not only with motor function (Park 2017). Quality of life of children with dystonia, may be improved by applying physiotherapy and rehabilitation approaches that support gross motor function.

Examining activities of daily living in individuals with disabilities and investigating the impact of independence in these activities on participation is highly valuable. In a study conducted with children with dyskinetic cerebral palsy, it was concluded that dystonia had a greater effect on activity compared to choreoathetosis and that dystonia emerging in the proximal regions of the extremities had a more detrimental impact on activity (Monbaliu et al. 2017). In our study, daily living skills such as self‐care, transfers, and communication were assessed using the WeeFIM, and strong associations were found between WeeFIM scores and quality of life. Some of this relationship may reflect broader aspects of general functional status and global disability rather than independence in daily activities alone (Park 2017). In children and adolescents with dystonia, continuous and involuntary contractions may restrict daily living skills such as self‐care, transfers, and communication, thereby negatively affecting quality of life. For this reason, interventions aimed at improving functionality and daily living skills may be incorporated into physiotherapy and rehabilitation programs for children with dystonia, and the use of technological support may also be considered.

This study has several limitations. First, the study sample included a relatively small number of children with dystonia with a wide age range and different etiologies. In addition, the findings of this study should be interpreted with caution due to the lack of generalizability to specific dystonia subgroups (e.g., by etiology or distribution). Future studies with larger sample sizes are needed to confirm the results. Second, the assessments used in this study were largely subjective in nature. Third, multiple statistical comparisons were conducted and this may increase the risk of Type I errors. Thus, the results should be interpreted with caution. Fourth, although several factors which may affect quality of life in children with dystonia were examined, other potential influences like comorbidities, socio‐environmental factors, or variations in treatment were not included. This may contribute to variability in outcomes. In addition to these limitations, the classification of the isolated and combined dystonia could not be determined due to limitations in the available dataset. This should be considered when interpreting the classification of dystonia subtypes. Finally, the children included in the study had varying levels of disease severity, including those with more severe motor and cognitive impairments, and the assessment tools used in this study required a certain degree of cooperation from the children. Even though children with a wide range of motor and cognitive levels were included, this requirement could have introduced a small degree of selection bias. Despite these limitations, the study provides valuable insights into factors influencing the quality of life in children with dystonia.

## 5 Conclusion

The findings of this exploratory study suggest that dystonia severity, motor functions, constipation status, and the level of independence in daily living activities may be associated with quality of life of children with dystonia. These results are giving a better understanding of potential factors contributing quality of life in this population. Future research may benefit from this study's findings especially planning interventions with a potential area of focus targeting gross motor function and daily living activities, while gastrointestinal‐related support may also be considered as a complementary approach. However, further research with larger and more homogeneous samples is needed to confirm these suggestions. In addition, researchers may consider assessing quality of life across different dystonia phenotypes, which could provide a better understanding to support individualized interventions.

## Author Contributions

**Kamile Uzun Akkaya**: conceptualization, methodology, investigation, writing – original draft, writing – review and editing, formal analysis, data curation. **Esra Serdaroglu**: conceptualization, methodology, writing – review and editing, resources, data curation. Pelin Atalan Efkere: conceptualization, methodology, investigation, data curation, writing – review and editing. **Zekiye Basaran**: conceptualization, methodology, data curation, writing – review and editing. **Bulent Elbasan**: conceptualization, writing – review and editing, supervision, resources.

## Funding

The authors have nothing to report.

## Ethics Statement

This study was performed in line with the principles of the Declaration of Helsinki. Approval was granted by the Ethics Committee of Gazi University (approval no: E‐77082166‐604.01.02‐300406, date: 02.22.2022).

## Consent

A signed parent‐informed consent form was obtained from the families of all children participating in the study.

## Conflicts of Interest

The authors declare no conflicts of interest.

## Data Availability

Research data are not shared.
